# Investigating the Impact of Benign Indication Hysterectomy on Pelvic Floor Symptoms and Sexual Function: A Prospective Study Integrating Pelvic Floor Ultrasonography and Surface Electromyography Test

**DOI:** 10.1177/26884844251379399

**Published:** 2025-09-22

**Authors:** Wanwen Chen, Kai Chen, Yan Wang, Yang Shen

**Affiliations:** ^1^ Department of Obstetrics and Gynaecology, Zhongda Hospital, School of Medicine, Southeast University, Nanjing, China.; ^2^ Novant Health Maternal Fetal Medicine, Winston-Salem, NC, USA.

**Keywords:** single-port laparoscopic, hysterectomy, female pelvic floor dysfunction, four-dimensional pelvic floor ultrasonography, pelvic floor sEMG test, female sexual dysfunction

## Abstract

**Objective:**

This prospective study aimed to investigate the short-term effects of single-port laparoscopic hysterectomy for benign conditions on postoperative pelvic floor symptoms, pelvic floor structure, and pelvic floor muscle function.

**Study Design:**

The study was conducted at Zhongda Hospital, Southeast University, from May 2022 to September 2023. Patients who underwent elective single-port laparoscopic hysterectomy for benign conditions were recruited. Paired *t*-tests and non-parametric rank-sum tests were used to compare changes in pelvic floor function, female sexual function index (FSFI), 4D pelvic floor ultrasonography, and surface electromyography (sEMG) indicators between pre- and postoperative periods.

**Results:**

A total of 71 participants were included, with 69 patients successfully followed up. There was a significant improvement in pelvic floor function postoperatively (*p* < 0.001). FSFI scores significantly decreased postoperatively. Bladder neck descent increased significantly postoperatively (*p* = 0.024) in 6 months. However, there were no significant differences in the other ultrasound indicators between preoperative and 6-month postoperative assessments. The time after the peak of the tonic contraction phase significantly decreased postoperatively (*p* = 0.047), and the average mean of post-baseline value decreased postoperatively with statistical significance (*p* < 0.001). There were no significant differences in other pelvic floor sEMG indicators between preoperative and postoperative assessments.

**Conclusions:**

Within 6 months post-hysterectomy, Pelvic Floor Disorders Impact Questionnaire-20 (PFDI-20) scores significantly improved, and FSFI scores significantly decreased postoperatively. Single-port laparoscopic hysterectomy did not significantly affect pelvic floor structure or muscle function in the short-term postoperative period. However, overall sexual function decreased within the same timeframe.

## Introduction

Hysterectomy is a prevalent therapeutic approach for benign gynecological conditions, extensively employed in treating uterine fibroids, endometriosis, and abnormal uterine bleeding.^1–3^ Pelvic floor disorders (PFD) constitute a heterogeneous group of conditions, encompassing urinary incontinence (UI), pelvic organ prolapse (POP), female sexual dysfunction (FSD), chronic pelvic pain, and fecal incontinence (FI), among others. UI, FI, and POP stand out as the predominant manifestations within the spectrum of PFD.^4^ Hysterectomy is extensively employed in the management of benign gynecological conditions. However, its effects on pelvic floor function in patients remain unclear. There is a paucity of research utilizing objective indicators to assess postoperative changes in pelvic floor structure and muscle function.

Pelvic floor ultrasound is characterized by real-time simplicity and high accuracy, enabling the visualization of structural changes in the pelvic floor before the manifestation of clinical symptoms, thereby facilitating preventive measures. Morphological changes in the levator ani muscles may affect pelvic organ support, with muscle contractility playing a crucial role in maintaining control^5^ While vaginal examination of pelvic floor muscles (PFMs) is a common method for assessing pelvic floor muscle function in physical therapy, pressure measurement methods have been found to be more reliable and effective in measuring and distinguishing muscle strength.^6,7^ Available quantitative methods include pressure measurement, surface electromyography (sEMG), ultrasound examination, and magnetic resonance imaging, among which sEMG is the most commonly used method in clinical practice.^6^ sEMG is an objective, non-invasive, and safe tool widely used for providing muscle feedback in daily physical therapy and monitoring.^7,8^

The present study aimed to evaluate short-term changes in pelvic floor function, anatomy, and muscle function in women undergoing total hysterectomy for benign conditions by integrating objective physiological indicators with subjective symptoms. This approach facilitated a more accurate and comprehensive assessment of pre- and postoperative pelvic floor function.

## Materials and Methods

### Study population

Patients undergoing elective hysterectomy for benign conditions at Zhongda Hospital, affiliated with Southeast University, from May 2022 to September 2023 were recruited for the study. The inclusion criteria encompassed patients undergoing concurrent adnexal surgery and those with adnexal masses (≤10cm) undergoing concurrent mass excision, with preoperative ultrasound assessment. Our center has extensive experience in single-port laparoscopic surgery, and all enrolled patients opted for this surgical approach after a comprehensive evaluation.

Patients were included in the study if they met the following criteria: (1) age between 40 and 64 years old and provided informed consent and (2) clinically indicated for hysterectomy within the fascia, including concurrent unilateral/bilateral salpingectomy, unilateral/bilateral oophorectomy, unilateral/bilateral adnexectomy, performed *via* single-port laparoscopic approach. Patients meeting any of the following criteria were excluded during screening: (1) preoperative diagnosis of POP, UI, or clinically indication for concurrent surgical treatment, (2) history of prior surgery for diagnosed PFD, (3) history of gastrointestinal surgery (excluding: endoscopy, hemorrhoidectomy/banding, *etc.*), pelvic/abdominal surgery (excluding: bilateral tubal ligation, intrauterine device removal/insertion, painless abortion/dilation and curettage, benign adnexal tumor resection, hysteroscopy, cervical polypectomy, cervical conization, *etc.*), (4) received hormone replacement therapy within the past year, (5) history of urological surgery within the past six months, (6) presence of significant cardiovascular or cerebrovascular disease, or requiring approval for major surgery, (7) patients with severe hemorrhoids, vaginitis, or urinary tract infection, (8) history of diagnosed malignant tumors, (9) history of major psychiatric disorders such as autism, intellectual disabilities, cerebral palsy, schizophrenia, bipolar disorder, *etc.*, and (10) deemed unsuitable for participation by the researchers. According to the inclusion and exclusion criteria, patients who met any of the following conditions were further excluded: (1) presence of severe complications graded IV-V according to the Clavien–Dindo classification system, (2) failure to meet the inclusion criteria but included mistakenly; (3) Incomplete data affecting evaluation, (4) postoperative occurrence of diseases in the urinary, nervous, digestive, or reproductive systems requiring medical or surgical treatment, (5) Postoperative pathological upgrading necessitating additional surgical or adjuvant therapy such as radiotherapy or chemotherapy, (6) Implementation of postoperative prophylactic PFM training, (7) Loss to follow-up after surgery, and (8) patient’s voluntary withdrawal from the study.

Patients included in this study underwent both 4D pelvic floor ultrasonography and pelvic floor sEMG. Additionally, they completed two questionnaire surveys: the Pelvic Floor Disorders Impact Questionnaire-20 (PFDI-20) and the female sexual function index (FSFI). Preoperative questionnaires were administered during face-to-face interviews, while postoperative questionnaires were conducted *via* telephone follow-up at 6 months postoperatively. Participants who couldn’t be reached after three attempts on different days were considered lost to follow-up. Pelvic floor sEMG data were collected and analyzed by the same professional, while 4D pelvic floor ultrasonography data were acquired by two specialized sonographers. All surgeries were performed by the same primary surgeon with the same assistant. Surgical data were recorded based on surgical records, and pathological types were documented based on postoperative pathological diagnoses.

### Ethics

This study has received approval from the Clinical Research Ethics Committee of Zhongda Hospital, affiliated with Southeast University, with approval number 2022ZDSYLL106-Y01.

### Assessments

#### 4D Pelvic floor ultrasound

The following parameters were predominantly incorporated into the ultrasound indices of this study: bladder neck descent (BND) (cm): bladder neck position (BNP) is measured as the distance from the bladder neck to the horizontal line of the lower edge of the symphysis pubis, both at rest and during the Valsalva maneuver. BND (cm) = BNP at resting sate-BNP during the Valsalva maneuver; urethral rotation angle (URA) (°): urethral tilt angle (UTA) is measured at rest and during the Valsalva maneuver, calculated as URA (°) = UTA during the Valsalva Maneuver-UTA at resting state; levator hiatus area (LHA) during Valsalva in 4D imaging (cm^2^); posterior vesicourethral angle (PVA): Measurement of the angle between the posterior wall of the bladder and the proximal urethra during the Valsalva maneuver. The symptoms of pelvic floor dysfunction are associated with structural changes in the anterior, middle, and posterior pelvic floor, which can be visualized through the use of anatomical monitoring of pelvic floor ultrasound.^9^

#### Pelvic floor sEMG

The specific acquisition steps of the sEMG included the following: (1) a 60-second pre-rest test to assess the function of the PFMs in the resting state; (2) five rapid contractions (each rapid contraction was followed by 10 seconds of rest) to assess the function of the fast PFMs. (3) Five sustained contractions and relaxations (10 seconds of contraction, 10 seconds of relaxation) were conducted to assess the function of the fast and slow PFMs. (4) A sustained 60-second contraction was performed to assess the function of the slow pelvic floor muscle. (5) A 60-second post-contraction assessment was conducted to evaluate the recovery function of the slow PFMs. (6) A resting test was conducted after 60 seconds to assess the recovery function of the PFMs. The following parameters were assessed: average mean of pre-baseline (μV); variability of pre-baseline; average peak of phasic contraction (μV); time after peak of phasic contraction (s); average mean of tonic contraction (μV); variability of tonic contraction; time after peak of tonic contraction (s); average mean of endurance contraction (μV); variability of endurance contraction; average mean of post-baseline stage (μV); and variability of post-baseline.^10–12^

#### PFDI-20

The PFDI-20 questionnaire is a validated subjective symptom assessment tool internationally recognized for evaluating the distress and severity of symptoms related to pelvic floor dysfunction. It comprises several subscales: the Pelvic Organ Prolapse Distress Inventory 6 (POPDI-6), Colorectal-Anal Distress Inventory-8 (CRADI-8), and Urinary Distress Inventory 6 (UDI-6).^13^ Consisting of 20 questions, each initiated with a “yes” or “no” response, the questionnaire prompts patients to rate the severity of bowel, bladder, or pelvic symptoms over the past three months using a 4-point scale. Scores are then transformed to a total score ranging from 0 to 300, with higher scores indicating greater symptom severity. Assessment metrics include the total score (points), POPDI-6 (points), CRADI-8 (points), and UDI-6 (points).

#### FSFI

The FSFI is a questionnaire consisting of 19 items designed to assess various aspects of sexual desire, arousal, lubrication, orgasm, satisfaction, and pain. FSFI is a globally validated multidimensional self-report questionnaire for evaluating sexual symptoms.^14^ The total score is calculated by multiplying the scores of six subscales by their respective predefined factors. Higher FSFI scores indicate better sexual function. Evaluation parameters include total score (points), sexual desire (points), subjective arousal (points), vaginal lubrication during sexual activity (points), orgasm (points), sexual satisfaction (points), and pain during intercourse (points).

### Descriptive statistics

Statistical analysis was performed using SPSS 26.0. The normality of continuous variables was assessed using the K-S test. For non-normally distributed continuous variables, results are presented as median and interquartile range (IQR), while normally distributed continuous variables are presented as mean and standard deviation (SD). Categorical variables are reported as counts with percentages.

### Preoperative and 6-Month postoperative comparative analysis

For both questionnaires (PFDI-20 and FSFI), scores for each subscale and the total score of the questionnaire were calculated for each sample using the calculation formulas described above. Data from 4D pelvic floor ultrasound and pelvic floor sEMG were recorded based on examination results. All included indicators were quantitative data. Statistical analysis was conducted using SPSS 26.0. Paired *t*-tests were utilized for normally distributed data, while Wilcoxon signed-rank tests were employed for non-normally distributed data to assess the differences in pelvic floor function before and after treatment. A significance level of *p* < 0.05 indicated statistical significance.

## Results

### Characteristics

**FIG. 1.**
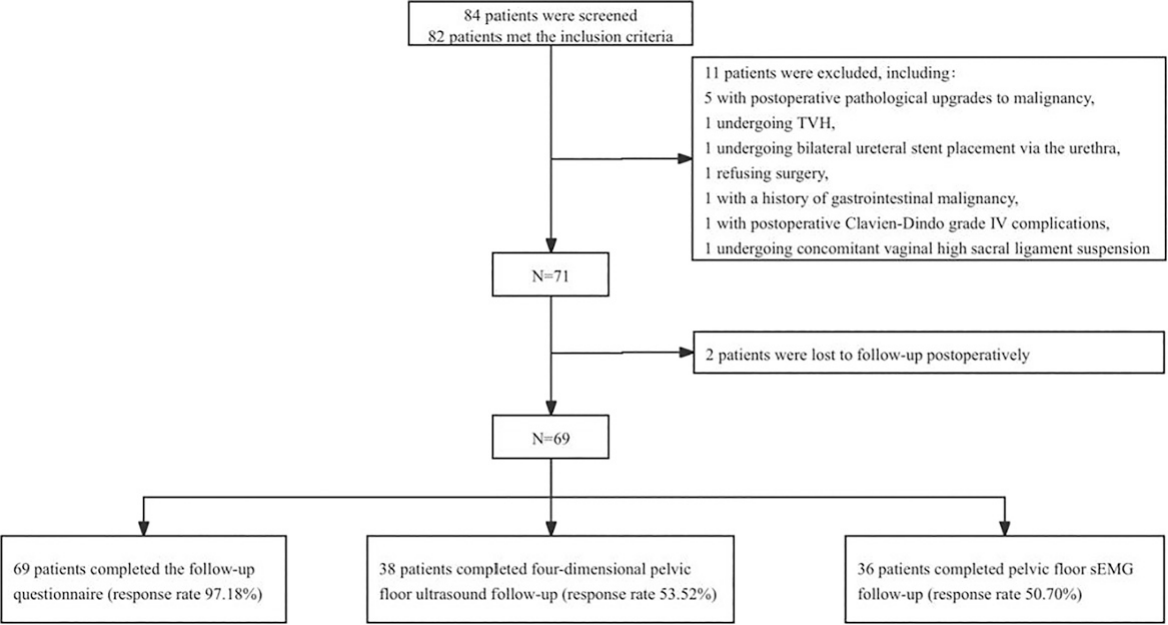
Flowchart: Screening process of the 69 participants included in the study and the number of completed follow-up questionnaires, pelvic floor sEMG, and four-dimensional pelvic floor ultrasound. sEMG, surface electromyography.

Eighty-four patients were screened, of whom 82 met the inclusion criteria. Subsequently, 11 patients were excluded due to exclusion criteria. Two patients were lost to follow-up postoperatively, resulting in a final inclusion of 69 patients in this study, as shown in Figure 1. Of the 69 participants, 35 completed assessments for the questionnaires, ultrasound, and sEMG; one participant completed the questionnaires and sEMG; three participants completed the questionnaires and ultrasound assessments; and the remaining 30 participants completed only the questionnaires.

As indicated in Table 1, the demographic profile of patients predominantly comprised middle-aged females, with the majority (66.7%) being premenopausal. Only one patient (1.4%) had no prior history of childbirth, and among those with a childbirth history, 88.4% had undergone vaginal delivery. No patients enrolled in this study had a history of smoking. 80.87% of participants underwent surgical intervention for uterine fibroids/adenomyosis, while approximately one-third underwent surgery for cervical lesions. Moreover, all patients who underwent bilateral oophorectomy were postmenopausal females.

**Table 1.** Baseline Characteristics of the 69 Patients Undergoing Hysterectomy Enrolled

Variables	Characteristics *N* = 69
	Mean (±SD)
Ages (years)	51.42 (±6.06)
Waist-to-hip ratio	0.87 (±0.06)
	Median (IQR)
Uterine weight (g)	238.1 (119.2,350.5)
Intraoperative blood loss (mL)	50 (50,200)
Postoperative length of hospital stays (days)	4 (4,5)
	Numbers (%)
BMI (body mass index, kg/m^2^)	
<18.5	1 (1.4)
18.5≤ <25	51 (74.0)
25≤ <30	15 (21.8)
30≤ <35	1 (1.4)
≤35	1 (1.4)
Parities	
0	1 (1.4)
1	52 (75.4)
2	10 (14.5)
3	6 (8.7)
Vaginal deliveries	
0	8 (11.6)
1	46 (66.7)
2	9 (13.0)
3	6 (8.7)
Cesarean sections	
0	61 (88.4)
1	8 (11.6)
Menopause	
Yes	23 (33.3)
No	46 (66.7)
Level of education	
Elementary school and below	32 (46.4)
High school and vocational school	22 (31.9)
College and above	15 (21.7)
Smoke	
Yes	0 (0)
No	69 (100)
Chronic diseases	
Hypertension	14 (20.3)
Diabetes mellitus	6 (8.7)
Systemic lupus erythematosus	1 (1.4)
Coronary heart disease	1 (1.4)
Asymptomatic hematuria	1 (1.4)
Sinus tachycardia	1 (1.4)
Rheumatoid arthritis	1 (1.4)
Nephrotic syndrome	1 (1.4)
Occupation type	
Employee	35 (50.7)
Retired employee	23 (33.3)
Unemployed	6 (8.7)
Teacher	2 (2.9)
Farmer	2 (2.9)
Worker	1 (1.4)
Nature of work	
Sedentary	40 (58)
Standing	15 (21.7)
Sedentary and standing	10 (14.5)
Free movement	4 (5.8)
History of pelvic surgery	
No	54 (78.3%)
Yes	
Appendectomy	7 (10.1)
Myomectomy	3 (4.3)
Unilateral salpingectomy	1 (1.4)
Unilateral oophorectomy	1 (1.4)
Unilateral ovarian cystectomy	2 (2.9)
Bilateral tubal ligation	1 (1.4)
Pathology	
Cervical lesions	22 (31.9)
Uterine fibroids	23 (33.3)
Uterine fibroids + adenomyosis	12 (17.4)
Endometrial atypical hyperplasia	5 (7.2)
Adenomyosis	7 (10.1)
Surgical procedure (besides hysterectomy)	
Unilateral salpingectomy	0 (0)
Bilateral salpingectomy	14 (20.3)
Unilateral tubal ligation	3 (4.3)
Bilateral tubal ligation	44 (63.8)
Duration of postoperative indwelling urinary catheter (days)	
1	1 (1.4)
2	11 (15.9)
3	52 (75.4)
4	2 (2.9)
5	2 (2.9)
6	1 (1.4)
Postoperative anal exhaustion time (days)	
1	16 (23.2)
2	45 (65.2)
3	4 (5.8)
4	3 (4.3)
6	1 (1.4)
Clavien–Dindo	
0	58 (84.1)
I	9 (13.0)
II	2 (2.9)

Age was defined as the patient’s age at the time of surgery. Uterine weight was measured immediately after dissecting uterine specimen’s *ex vivo*. For patients undergoing cervical cone biopsy or cold knife conization within 7 days prior to surgery, the weight of the cervix was included. For patients undergoing cervical loop electrosurgical excusion procedure (LEEP) or cold knife conization more than 7 days before surgery, the weight of the cervix was not included. The weight of specimens from cervical punch biopsy procedures was disregarded. Days were counted, and any duration of less than 24 hours was considered as one day. All patients included in this study began mobilization on the first day after surgery.

IQR, interquartile range; LEEP, loop electrosurgical excision procedure; SD, standard deviation.

### Differential analysis of preoperative and postoperative four-dimensional pelvic floor ultrasound and pelvic floor sEMG

There was a significant increase in the BND of patients at 6 months postoperatively, with a mean of 2.19 cm (SD 0.88), compared to the preoperative mean of 1.96 cm (SD 0.83) (*p* = 0.024). No significant statistical differences were observed between preoperative and 6-month postoperative URA, Valsalva state LHA, and PVA (Table 2).

**Table 2.** Pelvic Floor Structural Evaluation of Patients Undergoing Hysterectomy Preoperatively and at 6 Months Postoperatively Using Four-Dimensional Pelvic Floor Ultrasound

	Preoperatively	Postoperatively	
	Mean (±SD)	Mean (±SD)	*P*
BND	1.96 (±0.83)	2.19 (±2.19)	0.024^a^
URA	46.87 (±28.58)	45.32 (±45.32)	0.649
Valsalvastate PVA	133.42 (±18.74)	133.21 (±25.06)	0.948
	Median (IQR)	Median (IQR)	
Valsalva state LHA	20.74 (16.99, 25.60)	21.53 (16.65, 26.26)	0.587

^a^ Represents statistical significance. Data conforming to a normal distribution were denoted by Mean (SD), while non-normally distributed data were indicated by Median (IQR).

BND, bladder neck descent; LHA, levator hiatus area; PVA, posterior vesicourethral angle; URA, urethral rotation angle.

Table 3 showed that the time after the peak of tonic contraction decreased at 6 months postoperatively compared to preoperative values (*p* = 0.047). This suggests an improvement in the relaxation ability of pelvic floor slow muscles. Compared to that in preoperative, the average mean of post-baseline decreased postoperatively (*p* < 0.001), indicating an improvement in the hypertonic state of PFMs after contraction in the relaxation phase at 6 months postoperatively. However, the average mean of pre-baseline preoperatively was the same as at 6 months postoperatively (*p* = 0.228), indicating insufficient evidence of improvement in the hypertonic state of PFMs at rest postoperatively. No differences were observed in sEMG measurements between preoperative and 6-month postoperative assessments in the pre-baseline and phasic contraction, indicating no significant differences in the stability of PFM at rest and the function of pelvic floor fast muscles. Moreover, no differences were observed in the endurance contraction and variability of the tonic contraction phase and endurance contraction, suggesting no significant changes in the stability and contraction function of slow muscles postoperatively.

**Table 3.** Pelvic Floor sEMG in Patients Undergoing Hysterectomy Preoperatively and at 6 Months Postoperatively

	Preoperatively	Postoperatively	
	Median (IQR)	Median (IQR)	*P*
Pre-baseline			
Average mean (μV)	4.82 (2.70, 6.97)	4.41 (2.0, 5.69)	0.228
Variability	0.12 (0.11, 0.14)	0.12 (0.11, 0.14)	0.753
Phasic contraction			
Maximum (μV)	26.51 (14.41, 34.85)	27.26 (14.44, 38.12)	0.683
Time after peak(s)	0.33 (0.17, 1.05)	0.26 (0.19, 1.20)	0.481
Tonic contraction			
Average mean (μV)	13.77 (9.58, 20.75)	16.09 (15.4, 22.78)	0.974
Time after peak (s)	2.50 (0.51, 5.47)	1.66 (0.38, 4.9)	0.047^a^
Endurance contraction			
Average (μV)	11.62 (8.25, 19.59)	12.44 (9.58, 18.15)	0.961
Variability	0.22 (0.14, 0.32)	0.18 (0.14, 0.26)	0.164
Post-baseline			
Variability	0.12 (0.11, 0.18)	0.12 (0.11, 0.14)	0.588
	Mean (±SD)	Mean (±SD)	
Tonic contraction			
Variability	0.28 (±0.08)	0.26 (±0.08)	0.204
Post-baseline			
Average mean (μV)	5.02 (±2.95)	4.80 (±2.94)	<0.001^a^

^a^ Represents statistical significance. Data conforming to a normal distribution were denoted by Mean (SD), while non-normally distributed data were indicated by Median (IQR).

### Differential analysis of preoperative and 6-month postoperative PFDI-20 and FSFI

In our study, scores for POPDI, CRADI-8, UDI-6, and PFDI-20 total all decreased (PFDI-20P < 0.001, POPDI-6P < 0.001, CRADI-8 = 0.047, UDI-6P < 0.001), indicating improvements in participants’ reported pelvic floor symptoms (including pelvic discomfort symptoms and POP symptoms), urinary symptoms, and FI symptoms compared to preoperative levels (Table 4).

**Table 4.** Assessment of Pelvic Floor Dysfunction Symptoms in Patients Undergoing Hysterectomy Preoperatively and at 6 Months Postoperatively Using the PFDI-20

	Preoperatively median (IQR)	Postoperatively median (IQR)	*P*
POPDI-6	3 (0, 4)	0 (0, 4)	<0.001^a^
CRADI-8	0 (0, 2)	0 (0, 2)	0.047^a^
UDI-6	6 (1, 7)	3 (0, 3)	<0.001^a^
PFDI-20 total score	34.38 (20.83, 50)	20.83 (8.33, 29.17)	<0.001^a^

^a^ Represents statistical significance.

CRADI-8, Colorectal-Anal Distress Inventory-8; POPDI-6, Pelvic Organ Prolapse Distress Inventory 6; UDI-6, Urinary Distress Inventory 6.

Table 5 showed that the baseline median FSFI score for participants was 69 points (IQR 3, 84) preoperatively, which decreased to 56 points (IQR 2, 75) at 6 months postoperatively (*p* < 0.001). Additionally, scores across these six aspects were all decreased: sexual desire (*p* < 0.001), sexual arousal (*p* < 0.001), sexual lubrication (*p* < 0.001), sexual orgasm (*p* < 0.001), sexual satisfaction (*p* = 0.004), and sexual pain (*p* = 0.019). This suggests an overall decline in sexual function and various aspects of sexual function at 6 months postoperatively.

**Table 5.** Evaluation of Sexual Function in Patients Undergoing Hysterectomy Preoperatively and at 6 Months Postoperatively Using the Female Sexual Function Index

	Preoperatively median (IQR)	Postoperatively median (IQR)	*P*
Desire	4.2 (3.6, 4.8)	3.6 (3.6, 4.2)	<0.001^a^
Arousal	5.1 (4.5, 6.0)	4.2 (3.6, 4.8)	<0.001^a^
Lubrication	4.8 (4.2, 5.1)	3.9 (3.0, 4.8)	<0.001^a^
Orgasm	5.6 (5.2, 6.0)	4.8 (4.0, 5.6)	<0.001^a^
Satisfaction	4.8 (4.0, 5.6)	4.8 (3.6, 4.8)	0.004^a^
Pain	6.0 (4.8, 6.0)	6.0 (3.6, 6.0)	0.019^a^
Total	82 (71, 86)	74 (61, 78)	<0.001^a^

^a^ Represents statistical significance.

The average FSFI score <26 indicates FSD. In this study, 36.2% of patients reported an FSFI score <26 at the preoperative follow-up, all of whom were sexually inactive females. Among them, three individuals were divorced/widowed and had no sexual partners, one person reported fear of sexual activity due to continuous HPV infection, and the remaining reported lack of sexual desire either personally or from their partners. Among sexually active women before surgery, only one had a score <26 (FSFI score of 5). For women with preoperative scores below 26, all maintained postoperative scores below 26. Among those with preoperative scores exceeding 26, six exhibited postoperative scores below 26. After excluding patients who did not engage in sexual activity preoperatively, a differential analysis of FSFI scores was conducted for patients who engaged in sexual activity preoperatively (N = 45). The results showed a decrease in FSFI total scores postoperatively (median preoperative FSFI score 82, median postoperative FSFI score 74, *p* < 0.001), with scores in all six aspects of sexual function also decreased with statistical significance (Supplementary Table S1).

## Discussion

In our study of 69 patients undergoing hysterectomy for benign conditions, we observed improvements in pelvic floor symptoms, urinary symptoms, and colorectal symptoms within 6 months postoperatively. However, we noted a decline in sexual function at 6 months post-hysterectomy. Regarding pelvic floor structure, we observed changes only in the BND compared to preoperative values, while parameters such as URA, Valsalva state, LHA, and PVA had no differences. In terms of pelvic floor muscle function assessment, we found an improvement in the relaxation ability of pelvic floor slow muscles compared to preoperative values. The pelvic floor muscle hypertonicity observed during the relaxation phase after pelvic floor muscle contraction improved in patients at 6 months postoperatively.

The changes observed in pelvic floor symptoms before and after this study align with findings from prior perspective research. Through questionnaire surveys, it was noted that overall pelvic floor symptoms improved in participants six months postoperatively.^15^ Previous observational studies have also indicated that pelvic discomfort symptoms tend to ameliorate following hysterectomy during short-term follow-up assessments.^16^ Karjalainen et al. reported that postoperative UI symptoms were positively associated with factors such as larger uterine weight, presence of uterine fibroids, and lower body mass index (BMI) after hysterectomy.^17^ In our study, the majority (80.87%) of patients underwent surgery due to uterine fibroids/adenomyosis, with a median uterine weight of 238.1 g (IQR 119.2, 350.5). Surgical treatment, including hysterectomy, improves urinary symptoms post-fibroid treatment.^18^ Factors influencing pelvic floor function post-hysterectomy are multifaceted, with variables like multiple vaginal deliveries, obesity, and postoperative lifestyle habits playing significant roles. Previous studies also suggested that minimally invasive surgical approaches are deemed to facilitate faster postoperative recovery with minimal impact on pelvic floor function.^19^

The minimum clinically important difference (MCID) represents the smallest clinically meaningful change in score.^20^ There is a paucity of standardized values for MCID for PFDI-20 and FSFI. In previous MCID studies for PFDI-20, values of. The aforementioned studies reported a 13.5% increase or 23% were yielded,^21^ 23,^22^ 24,^23^ and 45,^24^ respectively, among patients who underwent POP surgery. However, the other baseline characteristics of the included populations were not consistent. One study calculated an MCID of 2.1 for FSFI,^25^ and the mean decrease in total FSFI score after surgery in this study was 9.59, indicating a clinically significant change in FSFI. However, there is a paucity of studies on MCID for FSFI and PFDI-20, and MCID appears to be disease- or intervention-specific.^26^ Consequently, there is a need for studies defining MCID in different patient populations, which is currently not defined for hysterectomy patients. A number of studies have demonstrated that MCID is contingent upon baseline scores, with higher symptom burden necessitating a greater perceived change.^27,28^ In the present study, the mean preoperative MCID for the PFDI-20 was 36.81 (total score of 300), rendering it challenging to ascertain whether it is clinically meaningful when utilizing levels specific to the population of POP patients.

Previous study suggested that in sexually active women, a decline in sexual function was observed 3 years postoperatively, with significant deterioration in sexual arousal, vaginal lubrication during sexual activity, and orgasm, with no clear correlation with the surgical pathway.^13^ Our study found a decline in sexual function in both the entire cohort and sexually active patients at 6 months postoperatively. This is different compared to other previous studies, which indicated an improvement in female sexual function post-hysterectomy.^29^ The influence of age and menopausal transition on sexual function might be the cause of this difference, and consideration of patients’ hormone levels is crucial in research.

This study was also designed to evaluate the postoperative changes in pelvic floor function through alterations in important anatomical structures and pelvic floor muscle strength. The results showed that hysterectomy only affected BND at 6 months postoperatively, with a mean increase from 1.96 cm preoperatively to 2.19 cm postoperatively. Larger BND values are associated with a higher risk of stress urinary incontinence (SUI), but mid-urethral mobility has a higher predictive value for SUI.^30^ Although there is no unified cutoff value for BND to diagnose SUI, some studies suggest that BND >2.4 cm may indicate SUI,^31^ while others suggest BND >2.5 cm.^32^ In this study, the mean postoperative BND was 2.19 cm, which may still be within the normal range and has no significant impact on urinary symptoms in the short term.

URA, an indicator for urinary activity monitoring and predicting UI,^33,34^ had a preoperative mean of 46.87° and a postoperative mean of 45.32° in this study. There was a slight but not significant reduction in the range of changes before and after surgery when the cut-off value of 45° (sensitivity 67.2%, specificity 79.8%) was utilized.^31^ Both preoperative and postoperative mean values of Valsalva maneuver PVA in our study were below the cut-off. Although partial detachment of the posterior bladder wall may occur during hysterectomy, it does not involve opening the distal portion of the ureters, resulting in minimal impact on PVA during surgery. In addition to its relevance in diagnosing bladder prolapse, studies have found that PVA is associated with the occurrence of SUI, with a higher risk of SUI with larger PVA values.^35^ This study did not observe an increase in PVA in the short term after surgery, consistent with the symptoms reported by the patients themselves.

Valsalva state LHA has been confirmed to be associated with the occurrence of POP, with women with POP having a larger LHA compared to those without POP.^36–38^ The enlargement of the LHA and damage to the levator ani muscle fibers leading to UI.^39^ In this study, the preoperative median Valsalva state LHA was 20.74 (IQR: 16.99, 25.60) cm^2^, and the postoperative median Valsalva maneuver LHA at 6 months was 21.53 (IQR: 16.65, 26.26) cm^2^. There was no significant difference in LHA change in the short term after surgery compared to that before surgery. Our study suggested that changes in important pelvic floor anatomical structures after single-port laparoscopic hysterectomy did not lead to the development of PFD in the short term. Further study is needed to investigate changes in pelvic floor-related symptoms from other relevant factors.

sEMG can reflect female sexual function indirectly. Women with higher pelvic floor muscle strength report better sexual function outcomes, including desire, arousal, orgasm, and satisfaction.^40^ It has been found that sexually active women have significantly longer durations of pelvic floor muscle contraction compared to those who are not sexually active. Similarly, women who experience orgasms have significantly longer durations of pelvic floor muscle contraction.^41^ Currently, there is a lack of studies on the correlation between sEMG and overall pelvic floor function symptoms. Our study found synchronous improvements in PFDI-20 overall scores, individual subscale scores, and pelvic floor muscle relaxation ability after contraction. It suggested a possible correlation between pelvic floor muscle function recovery and postoperative improvement in pelvic floor symptoms.

This is a prospective study to observe changes in patients’ self-reported pelvic floor function before and after single-port laparoscopic hysterectomy. Four-dimensional pelvic floor ultrasound was also used to observe structural changes before and after surgery. sEMG suggested improvements in postoperative pelvic floor slow-twitch muscle relaxation, decreased contraction function, and improved pelvic floor muscle relaxation after contraction compared to preoperative levels. Combined with objective examination results, these findings may, at least partially, explain the reasons for patients’ subjective PFD symptom changes postoperatively.

The study has some limitations. Patients requiring pelvic floor reconstruction for POP and UI simultaneously were excluded, which might overlook the beneficial effects of hysterectomy in PFD, such as POP. The sEMG test in the study is a single, non-same-day measurement. Despite the use of various techniques to minimize potential measurement errors, the current clinical applications of the test may not fully guarantee its clinical validity.^42^ Furthermore, long-term, multi-timepoint follow-up studies are still needed to ascertain the long-term effects. Lastly, the study found improvements in pelvic floor symptoms but decreased sexual function postoperatively. Future research should include assessments of patients’ quality of life to explore whether surgical effects on pelvic floor function affect overall quality of life.

## Conclusion

Our study suggested short-term declines in female sexual function postoperatively. Patients' symptoms before surgery and their expectations of surgical management, including hysterectomy, should be evaluated carefully. In the assessment of anatomical structure and muscular function, no significant deterioration is evident in the short term, which can help alleviate patient concerns regarding postoperative outcomes in this regard. But outcomes of surgical options, including hysterectomy, only to treat patients who experience concurrent pelvic floor discomfort are still unclear and need further study.

## Abbreviations Used

BMI: Body Mass Index
BND: Bladder Neck Descent
BNP: Bladder Neck Position
CRADI-8: Colorectal-Anal Distress Inventory-8
FI: Fecal Incontinence
FSD: Female Sexual Dysfunction
FSFI: Female Sexual Function Index
IQR: Interquartile Range
LEEP: Loop Electrosurgical Excusion Procedure
LHA: Levator Hiatus Area
MCID: Minimum Clinically Important Difference
PDFI-20: Pelvic Floor Disorders Impact Questionnaire-20
PFD: Pelvic Floor Disorders
PFMS: Pelvic Floor Muscles
POP: Pelvic Organ Prolapse
POPDI-6: Pelvic Organ Prolapse Distress Inventory 6
PVA: Posterior Vesicourethral Angle
SD: Standard Deviation
SEMG: Surface Electromyography
SUI: Stress Urinary Incontinence
UDI-6: Urinary Distress Inventory 6
UI: Urinary Incontinence
URA: Urethral Rotation Angle
UTA: Urethral Tilt Angle
