# Supplementary material for: Investigating the Impact of Benign Indication Hysterectomy on Pelvic Floor Symptoms and Sexual Function: A Prospective Study Integrating Pelvic Floor Ultrasonography and Surface Electromyography Test
Source: Womens Health Rep (New Rochelle). 2025 Sep 22;6(1):945–55. doi: 10.1177/26884844251379399 (PMC12547404; doi:10.1177/26884844251379399)
Supplement: Supplementary Table S1 [file 26884844251379399_supplementary_table_s1.docx]

|  | Preoperatively  Median (IQR) | Postoperatively  Median (IQR) | P |
| --- | --- | --- | --- |
| Desire | 4.2(3.6,4.8) | 3.6(3.6,4.2) | <0.001* |
| Arousal | 5.1(4.5,6.0) | 4.2(3.6,4.8) | <0.001* |
| Lubrication | 4.8(4.2,5.1) | 3.9(3.0,4.8) | <0.001* |
| Orgasm | 5.6(5.2,6.0) | 4.8(4.0,5.6) | <0.001* |
| Satisfaction | 4.8(4.0,5.6) | 4.8(3.6,4.8) | 0.004* |
| Pain | 6.0(4.8,6.0) | 6.0(3.6,6.0) | 0.019* |
| Total | 82(71,86) | 74(61,78) | <0.001* |

**Table S1.** Evaluation of Sexual Function in Patients Undergoing Hysterectomy with Preoperative Sexual Activity Preoperatively and at 6 Months Postoperatively Using the FSFI. * represents statistical significance.
